# Robotic Stereotactic Ablative Radiotherapy for Patients with Early-Stage Lung Cancer: Results of an Interim Analysis

**DOI:** 10.3390/cancers16183227

**Published:** 2024-09-22

**Authors:** Anna Zygogianni, Ioannis M. Koukourakis, John Georgakopoulos, Christina Armpilia, Zoi Liakouli, Dimitra Desse, Georgios Ntoumas, Foteini Simopoulou, Maria Nikoloudi, Vassilis Kouloulias

**Affiliations:** 1 Radiation Oncology Unit, Aretaieion Hospital, School of Medicine, National and Kapodistrian University of Athens, 11528 Athens, Greece; ikoukourakis@med.uoa.gr (I.M.K.); ioangeo@med.uoa.gr (J.G.); charbilia@med.uoa.gr (C.A.); zliakouli@aretaieio.uoa.gr (Z.L.); dimidesse@gmail.com (D.D.); geontoumas@yahoo.com (G.N.); foteinisim@hotmail.gr (F.S.); mnikoloud@med.uoa.gr (M.N.); 2 Department of Clinical Radiation Oncology, Attikon Hospital, School of Medicine, National and Kapodistrian University of Athens, 12462 Athens, Greece; vkouloul@med.uoa.gr

**Keywords:** lung cancer, stereotactic radiotherapy, CyberKnife, radiobiology

## Abstract

**Simple Summary:**

In this article, we report the results of an interim analysis of 81 early-stage lung cancer patients undergoing stereotactic ablative radiotherapy with the CyberKnife M6 robotic radiosurgery system, with the endpoints being treatment efficacy and tolerance. Within a median follow-up of 20 months, a grade 2/3 lung toxicity of no clinical significance was reported in 6% of the patients. A higher biological effective dose and larger irradiation lung volumes were associated with increased toxicity. The projected 24-month local progression-free survival rate was 95%. These results confirm the safety and efficacy and further cement the role of robotic stereotactic ablative radiotherapy for early-stage lung cancer patients as an eventual alternative to surgery.

**Abstract:**

Background/Objectives: Surgery is the primary treatment for early-stage lung cancer. Patients with medically inoperable lung carcinomas and patients who refuse to undergo surgery are treated with definite radiotherapy. Stereotactic ablative radiotherapy (SABR) is a compelling non-invasive therapeutic modality for this group of patients that confers promising results. Methods: We report an interim analysis of an ongoing trial. Eighty-one patients with medically inoperable early-stage (T1,2N0) lung cancer underwent SABR in our institution. SABR was delivered via the CyberKnife M6 robotic radiosurgery system. The endpoints of the analysis were treatment efficacy and tolerance. Results: There were no acute or late toxicities from the skin or the connective tissue of the thorax. A grade 2/3 lung injury of non-clinical significance was noted in 6% of patients, which was directly related to a higher biologically effective dose (BED_α/β = 3_) and larger irradiation lung volumes in both univariate and multivariate analyses. A local control (LC) was achieved in 100% of the patients at the first follow-up, and the projected 24-month local progression-free survival (LPFS) rate was 95%. The projected 24-month disease-specific overall survival (OS) was 94%. Conclusions: High LC and OS rates can be achieved with SABR for early-stage lung cancer, with minimal toxicity. This study continues to recruit patients.

## 1. Introduction

Lung carcinoma is the second most common cancer in Greece, behind breast and prostate cancer, with 78.6 and 21.0 estimated cases per 100,000 men and women, respectively, per year; it is also the principal cause of cancer-related death [1,2]. Non-small cell lung cancer (NSCLC) represents approximately 85% of lung cancers [3]. Surgical treatment is the gold standard for early-stage NSCLC. However, patients with medically inoperable carcinomas due to poor performance status (PS) and/or severe comorbidities (e.g., chronic obstructive pulmonary disease—COPD—and cardiovascular disease) or patients that refuse to undergo surgery are treated with definitive radiotherapy (RT), with results comparable to surgery [4,5,6].

Moderately hypofractionated (2.5–4 Gy/f) 3D-RT schedules were originally investigated with promising data in terms of local control and toxicities [7,8]. The rapid advances in RT delivery systems, however, led to clinical trials that compared the efficacy of 3D-RT to stereotactic RT with large RT fractions (up to 20 Gy) [9,10], favoring the latter for the treatment of early-stage NSCLC. In these cases, RT is delivered using stereotactic techniques that allow increased dose accumulation to the target while sparing the surrounding normal tissues from the adverse effects of RT (stereotactic ablative radiotherapy—SABR). Although standard linear accelerators can be utilized towards this goal, the CyberKnife (CK) system, with its unique characteristics, can safely deliver ablative doses to lung tumors [11]. Similarly, studies have demonstrated favorable results of SABR with or without chemotherapy in cases of inoperable early-stage small cell lung cancer (SCLC) [12,13].

The long-term results of the STARS trial—a single-arm, prospective trial of early-stage lung cancer patients treated with SABR, with a pre-specified comparison to surgery—showcased that SABR confers comparable survival rates to lobectomy and mediastinal lymph node dissection [14]. A phase III randomized trial (VALOR) is ongoing, aiming to conclusively address this issue [15].

In this retrospective study, we report our preliminary experience with treating early-stage lung cancer using the CK M6 robotic radiosurgery system with a lung-optimized treatment (LOT) module (Accuray Inc., Madison, WI, USA). Local control (LC), local progression-free survival (LPFS), and disease-specific overall survival (OS) are analyzed. Treatment-related toxicities are reported, while future perspectives are discussed.

## 2. Materials and Methods

### 2.1. Patients

The current study reports an interim analysis of the results of SABR for histologically confirmed or clinically diagnosed medically inoperable early-stage lung cancer. Eighty-one patients underwent SABR with the CK M6 robotic radiosurgery system equipped with an InCise (2) Multileaf Collimator and with an LOT module, together with the real-time image-guided Synchrony Respiratory Tracking System (Accuray Inc., Madison, WI, USA) to deal with target movement, during the first 3 years after its installation in our institution. The primary and secondary endpoints were treatment efficacy and short- and long-term RT toxicity, respectively.

PET-CT was performed on all patients regardless of pathology status. Both central and peripheral lung cancers were treated. Radiological imaging demonstrated absence of metastatic disease in all patients. Each case was discussed by a panel of radiation oncologists, thoracic surgeons, and medical oncologists. The vast majority of patients were deemed inoperable due to comorbidities (COPD, cardiovascular disease) and poor PS. Specifically, 70 patients (86%) were considered high-risk for a surgical procedure, while 11 patients (14%) did not accept the choice of surgery. The local Ethics and Research Committee approved this retrospective study (no: 484/16-01-2023). Written and informed consent was acquired from all patients before treatment. All patients consented to the use of their treatment data for research purposes. The median follow-up (clinical and radiographic) was 20 months (3–38 months). Three patients were lost to follow-up immediately after therapy completion.

### 2.2. Treatment Planning

For each patient, four series of thoracic computed tomography (CT) images (slice thickness 1 mm, 512 × 512 pixels) for LOT planning were acquired (exhalation, inhalation, normal breathing, and normal breathing with IV contrast) using a Philips Brilliance 16 CT-sim scanner). Seven patients with deteriorated renal function did not receive IV contrast during simulation.

The Accuray Precision Treatment Planning Software (v.3.1.1, Accuray Inc., Madison, WI, USA) was used to generate treatment plans in conjunction with the LOT module and Xsight Lung Tracking system to compensate for translational lung motion during delivery. The gross tumor volume (GTV) and organs at risk (OAR) were identified and delineated via lung and mediastinum windows and also by using PET-CT fusion.

There was no margin left from GTV to clinical target volume (CTV), according to the RTOG trials for lung SABR. The CTV margins were expanded by 3–5 mm (depending on tumor proximity to critical structures) in the tracked direction and by 5–8 mm in the untracked direction to establish the planning target volume (PTV). The contoured OAR included the lungs, spinal cord, ribs, esophagus, trachea/bronchi, large vessels, and heart.

Plans were created by applying the Ray-Tracing Algorithm with lateral correction for heterogeneity and were optimized using the InCise Multileaf Collimator (Accuray Inc., Madison, WI, USA) The LOT module of the CK Xsight Lung Tracking System (Accuray Inc., Madison, WI, USA) allowed for the application of fiducial-free motion management strategies. During CK treatment delivery, the Synchrony Respiratory Tracking System (Accuray Inc., Madison, WI, USA) continuously synchronized treatment beam delivery to the motion of a target that was moving with respiration. Motion correlations of the internal tumor location on two images, acquired with two in-room orthogonal X-rays, can be estimated in order to create a synchrony correlation model for translational motion during treatment delivery, with the external respiratory signal arising from three light-emitting diodes (LEDs) being fixed on the patient thorax. Treatment with CK (including patient positioning and real-time imaging) lasted approximately 22 min per fraction. While longer treatment time per fraction may suggest potential uncertainties in patient intra-fraction motion when compared to standard linear accelerators (usually 12–15 min with cone beam computed tomography before RT initiation), real-time imaging and tracking of patient motion and breathing allows safe and effective delivery of RT. Further information regarding the equipment and treatment planning system of CK can be found at https://www.accuray.com/cyberknife-m6/ (accessed on 16 September 2024).

The three most common SABR treatment schedules were 20 Gy per fraction for a total of 60 Gy (15 patients), 18 Gy per fraction for a total of 54 Gy (27 patients), and 10 Gy per fraction for a total of 50 Gy (17 patients), prescribed at the 80% isodose line (Table 1). The maximum dose was defined by the 100% isodose line. The different RT schedules applied depended on the patient’s PS, tumor size, and tumor location. Specifically, 3 fractions of 20 or 18 Gy were prescribed for peripheral tumors, according to patient PS and tumor size (larger fractions for smaller tumors), while 5 fractions of 10 Gy were most commonly used for central tumors (located near major vessels and bronchi). Finally, the choice of fractionation was at the discretion of the physician.

### 2.3. Radiobiological Considerations

The linear-quadratic model is safely applied to fractionation doses up to 10 Gy. Although this model can also be used for higher doses per fraction similar to the ones in SABR [16], the validity of this model is questionable as vascular damage increases disproportionately, and immunological response parameters may differently affect the biological injury to cancer and normal tissues [17]. We have calculated the equivalent dose delivered in 2 Gy fractions (EQD2) using the following formula: EQD2 = Total dose × ([α/β + dose per fraction]/[α/β + 2]). The biologically effective dose (BED) was also calculated as follows: BED= Total dose x (1 + [dose per fraction/{α/β}]) (Table 1). For normal lung tissue radiation damage, we considered an α/β = 3 Gy [18], while for tumor tissue we considered an α/β = 10 Gy [19].

### 2.4. Treatment and Toxicity Evaluation

Blood tests were required before treatment initiation. Patients were monitored daily for potential acute adverse effects. A CT scan was performed at the 3-month time point after RT and every 3 months thereafter. A PET-CT scan was ordered 6 months after therapy completion and then annually unless there was evidence of loco-regional or distant relapse, as indicated in imaging or physical examination. Any type of response (complete-CR or partial-PR) or stable disease (SD) per the RECIST 1.1 criteria was documented 3 months after RT [20]. Complete or partial metabolic response and stable metabolic disease (PERCIST criteria) were also indicative of lack of progression [21]. The definition of local progressive disease (PD) was based on the aforementioned criteria (an increase in tumor size by more than 20% or an increase in the SUV peak of the FDG uptake by >30%). OS was defined as time after RT completion until death or at the time of last follow-up. LPFS describes the time between the completion of RT and the documentation of local progression.

Treatment toxicity was assessed during each follow-up. Acute and late adverse events, with the exception of radiation-induced lung toxicity (RLT), were graded using the Common Terminology Criteria for Adverse Events (CTCAE) v.5 version [22]. RLT was scored based on a grading scale that has already been proposed by our group in a previous study (Supplemental Table S1) [23].

### 2.5. Statistical Analysis

Statistical analysis and presentation of graphs were performed using the GraphPad Prism 8.0.2 version and IBM SPSS Statistics Version 26. The non-parametric Mann–Whitney or Kruskal–Wallis (with subsequent Dunn test) tests were used to compare groups of continuous variables, as appropriate. The impacts of age, PS, irradiated tumor volume (TVI), and BED_α/β = 3_ on toxicity score were analyzed using univariate and multivariate regression models. The impacts of age, PS, histopathology status, TVI, and BED_α/β = 10_ on LPFS were analyzed by using the Cox regression model in terms of univariate and multivariate analyses. Overall, all available patient data were used for the univariate regression models. Clinical factors that were shown to be statistically significant in univariate analysis were subsequently used for multivariate regression models [24]. Comparisons of different LPFS in terms of PS were performed by using the log-rank test with the Kaplan–Meier survival curves. All the analyses were performed with a *p*-value < 0.05 that was set for significance.

## 3. Results

Patient and disease characteristics are displayed in Table 2. The median age of the study participants was 73, and most patients (69/81–85%) had good PS (0,1). NSCLC concerned 50/51 of the pathology reports, while SCLC was histologically proven in only one case. In 30 patients, there was no histological confirmation (non-diagnostic biopsy), and the diagnosis was based on a PET-CT scan only. The median size of tumors was 24 mm.

### 3.1. Treatment Response and Survival

**Figure 1:**
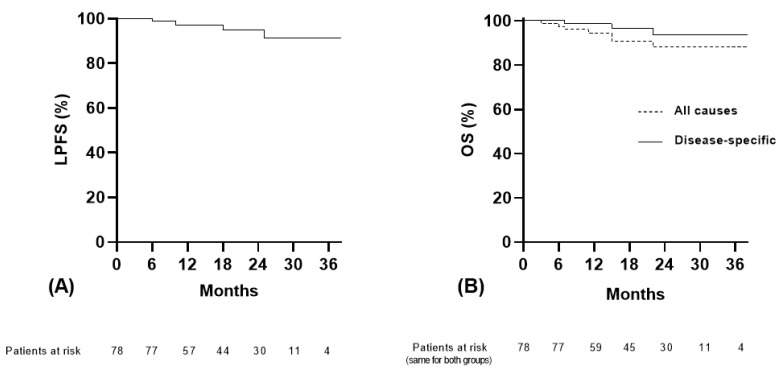
Kaplan–Meier survival curves: (**A**) local progression-free survival (LPFS) and (**B**) all-cause and disease-specific overall survival (OS).

**Figure 2:**
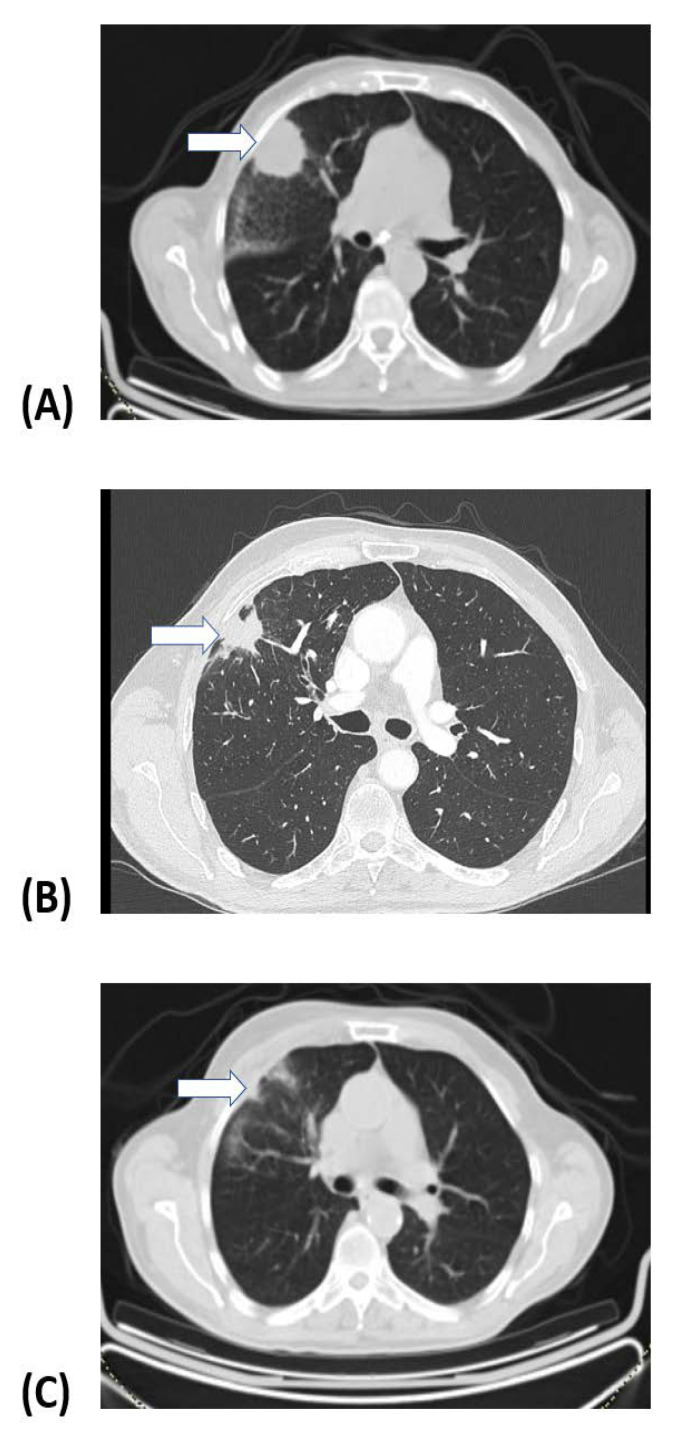
A patient with peripheral adenocarcinoma of the lung (arrow) treated with 3 fractions of 20 Gy (**A**). (**B**,**C**) show the tumor responses (arrows) 3 and 6 months after treatment completion.

None of the patients displayed disease progression 3 months after the RT completion. Overall, 44 (56%) patients achieved CR, 23/78 (30%) displayed PR, and 11 (14%) patients had SD. Within a median follow-up of 20 months, only four patients (5%) presented with local in-field disease progression. Figure 1A shows the Kaplan–Meier LPFS curves. The projected 24-month LPFS rate was 95%. Figure 2 shows a typical example of CR 6 months after SABR.

**Figure 3:**
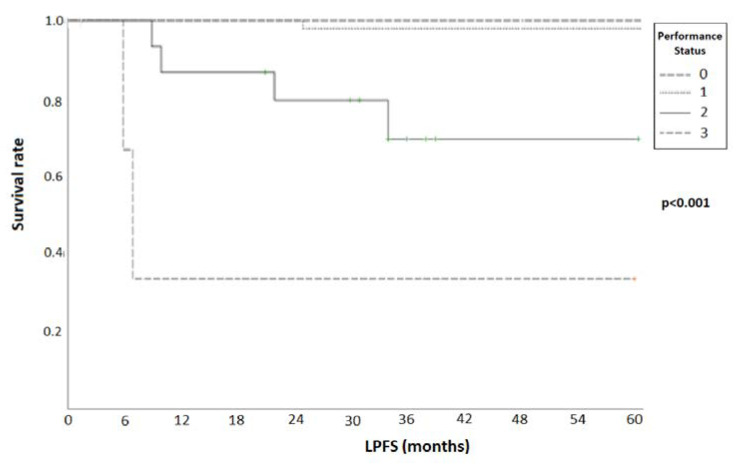
Kaplan–Meier curves for different performance statuses associated with local progression-free survival (LPFS) (*p* < 0.001, log-rank test).

In a univariate Cox regression analysis of different parameters that could potentially affect patient LPFS, a better PS and higher BED_α/β = 10_ were significantly linked with improved LPFS (*p* = 0.014 and 0.033, respectively). There was no significant association with age, histology, and TVI (*p* = 0.41, 0.89, and 0.21, respectively). In a subsequent multivariate Cox regression analysis, PS was the only factor with a significant correlation with LPFS (*p* = 0.003), while the BED_α/β = 10_ lost its significant impact (Table 3). There was a significant association, in terms of LPFS, when analyzing groups of patients with different PS, as shown in Figure 3 (log rank test, *p* < 0.001).

Out of 78 patients, 4 developed distant metastases. Metastasis was the cause of death for three of them, who also displayed local progression. The fourth patient developed distant metastases without local progression and was alive at the time of the last follow-up. Four additional patients died from intercurrent disease. Figure 1B displays the Kaplan–Meier disease-specific and all-cause OS curves. The projected 24-month disease-specific OS rate was 94%.

### 3.2. Treatment Tolerance

**Figure 4:**
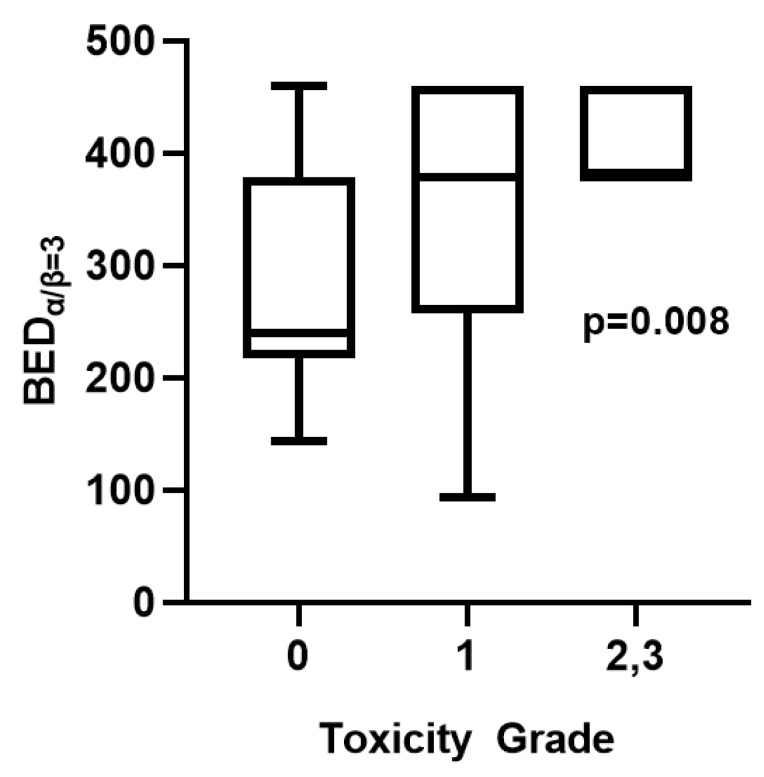
Correlation between the biologically effective dose (BED) calculated for α/β = 3 with lung toxicity grade (Kruskal–Wallis test: *p* = 0.008; Dunn test for multiple comparison: 0 vs. 1—*p* = 0.07, 0 vs. 2,3—*p* = 0.03, 1 vs. 2,3—*p* = 0.59).

There was no acute or late sequel from the skin and connective tissue (bones, muscles, and nerves) of the thorax. Localized lung toxicities, however, were frequently recorded in radiological examination, although of non-clinical significance. None of them, however, presented with clinical symptomatology. Within a median follow-up of 20 months, 73 out of 78 (94%) patients demonstrated minimal lung injury on their CT scans. Five patients (6%) developed grade 2 or 3 lung toxicity. No grade 4 toxicity was recorded. Figure 4 displays the BED_α/β = 3_ values for the three groups of lung toxicity (0 vs. 1 vs. 2,3; *p* = 0.008).

The mean value of the irradiated tumor volume (TVI) was 32.62 cc. In a univariate regression analysis of different parameters potentially affecting lung toxicity, a larger TVI and higher BED_α/β = 3_ were significantly associated with higher toxicity (*p* = 0.0001 and 0.0001, respectively) (Table 4). No association was noted between either age or PS and lung toxicity (*p* = 0.44 and 0.49, respectively). In a subsequent multivariate regression analysis, this significant association between TVI, BED_α/β = 3_, and toxicity was sustained (*p* < 0.0001 and 0.0001, respectively) (Table 4).

## 4. Discussion

SABR has been gaining more and more ground for medically inoperable early-stage lung cancer in light of results comparable to surgical resection [4,5,6]. An extensive meta-analysis comparing the efficacy of surgery and SABR has been published by Chen et al. [25]. COPD, cardiovascular disease, and refusal to undergo surgery render ablative RT a compelling, non-invasive treatment with high LC rates and acceptable toxicity. Several studies have been published reporting data on lung cancer patients treated with SABR [26]. In a report by Khadige et al., LC was achieved in 88% of patients at the 24-month time point for patients with primary lung cancer or pulmonary metastases receiving different SABR schedules [27]. In a more recently published retrospective study on 55 patients with stage I NSCLC who underwent SABR delivering a BED_α/β = 10_ higher than 100 Gy in 3, 5, or 8 fractions, 3-year OS and PFS rates were 82% and 77%, respectively [28]. Grade 2 radiation pneumonitis was observed in 14.5% of the study participants. In addition, Stanic et al. analyzed 206 early-stage lung cancer patients, the majority of whom received SABR with five fractions of 10–11 Gy [29]. The 5-year LPFS and OS rates were 82% and 31%, respectively, and 13% of patients displayed grade 1 and 2 toxicity. A phase II clinical trial on 65 patients with stage I NSCLC demonstrated low local and regional recurrence rates 7 years after SABR was delivered in four fractions of 12.5 Gy (8.1% and 13.6%, respectively) [30]. The 7-year OS rates were 47.5%, and 4.6% of patients developed grade 3 radiation pneumonitis. Finally, the results of the iSABR phase II non-randomized trial were recently published, and they included 217 patients with early-stage NSCLC or lung metastases from solid tumors [31]. Different SABR schedules were applied, varying from 1 to 8 fractions. LPFS rates at 5 years ranged from 83% to 93%, with grade 3–5 toxicity occurring in 5% of patients.

In this study, we have reported results from our experience in treating lung cancer patients with the CK system during a 38-month time period. Three months after RT completion, all patients displayed tumor regression or at least SD. The projected 24-month LPFS and disease-specific OS rates were promising. These data follow a similar pattern to the results already published in the aforementioned prospective and retrospective studies. Moreover, PS appeared to be an important predictor of loco-regional progression in the multivariate analysis. A similar association of PS with OS has been reported in a previous study by Kang et al. [32].

Grade 0,1 RLT was observed in 94% of patients, while grade 2 and 3 toxicity of non-clinical significance were noted in 6% of cases. Whether the levels of the biological radiation dose define lung toxicity in SABR is unclear. There is no conclusive evidence regarding the validity of the LQ model when delivering higher than 10 Gy fractions. Ablative (>10 Gy) radiation fractions lead to enhanced vascular injury, which in turn is responsible for indirect cell death. Augmented immunity against cancer is also a subsequent effect of SABR. These radiobiological events have raised questions about the LQ model and its applicability in high-dose/fraction RT schedules [33]. There are, however, studies on SABR and NSCLC that have proceeded to a radiobiological analysis of their results with the LQ model [16]. Therefore, we calculated the BED_α/β = 3_ of the different RT schedules we prescribed. In both univariate and multivariate analyses, the BED_α/β = 3_ was directly associated with lung toxicity. A similar association was also noted for larger TVIs. Cases et al. recently reported a retrospective analysis of the lung toxicity of 102 NSCLC patients who underwent SABR and showcased that a BED_α/β = 3_ higher than 300 Gy is associated with a higher incidence of organizing pneumonia [34].

Several limitations of this study have to be taken into consideration. A larger number of patients and longer follow-ups are required in order to extract safer conclusions. However, these early results have already successfully adhered to the published data. Moreover, different SABR schedules were utilized to treat patients. A future analysis, though, on the different BEDs and their subsequent effects on RLT and LC, in combination with a larger cohort of patients, could potentially shed light on the radiobiological mechanisms underlying normal and lung cancer tissue injury when delivering ablative RT doses.

Another limitation is the absence of histological confirmation in 30/78 patients. This could have positively affected the results of our study, as one can argue that non-malignant lesions were unknowingly treated. Nevertheless, the small size of lesions and varying locations that render the biopsy procedure difficult often lead to non-diagnostic results. In a study by Borelli et al., non-diagnostic results for lesions less than 20 mm occurred in approximately 31% of patients [35]. A recent retrospective analysis comparing the effects of SABR on 131 patients with a clinical diagnosis of lung cancer and 131 patients with an available pathology report demonstrated no significant differences in terms of recurrence or survival between the two groups [36]. It can be suggested that patients with a lung cancer-indicative PET-CT scan can be safely treated with SABR regardless of the availability of a pathological confirmation.

The recent impressive results of a randomized phase II trial, reporting a 24% improvement in event-free survival rates with the addition of nivolumab to SABR for stage I-II NSCLC, further cements the role of SABR in the treatment of early-stage lung cancer [37]. Randomized trials of immuno-SABR vs. surgery should be thoroughly examined in the future.

## 5. Conclusions

In conclusion, the current interim analysis suggests that SABR for early-stage lung cancer confers promising results as far as LPFS and OS are concerned, with minimal toxicity. A higher BED and larger TVI appear to be associated with increased toxicity, which could be considered in clinical practice. This study is ongoing to recruit a larger number of patients with a longer follow-up.

## Figures and Tables

**Table 1:** Treatment schedules and radiobiological analysis.

Fractions/ Dose per Fraction	No. Pts	EQD2 (Gy) *	EQD2 (Gy) **	BED (Gy) *	BED (Gy) **
3/15 Gy	1	162	93.8	270	112.5
3/17 Gy	2	204	114.8	340	137.7
3/18 Gy	27	226.8	126	378	151.2
3/20 Gy	15	276	150	460	180
4/12 Gy	4	144	88	240	105.6
5/10 Gy	17	130	83.3	216.7	100
5/11 Gy	4	154	96.3	256.7	115.5
5/12 Gy	2	180	110	300	132
6/8 Gy	1	105.6	72	176	86.4
7/6.5 Gy	1	86.5	62.6	144.1	75.1
8/7.5 Gy	4	112	79.3	186.7	95.2
10/4 Gy	1	56	46.7	93.3	56
12/5 Gy	1	96	75	160	90
13/3.5 Gy	1	59.2	51.2	98.6	61.5

(*) For α/β = 3 Gy without time correction. (**) For α/β = 10 Gy without time correction. Abbreviations: pts = patients; EQD2 = equivalent dose delivered in 2 Gy fractions; BED = biologically effective dose.

**Table 2:** Patient and disease characteristics.

No Pts	81	%
PS		
0	16	20
1	53	65
2	11	14
3	1	1
Gender		
Male	54	67
Female	27	33
Age		
Median	76	
Range	47–92	
Histology		
Squamous	24	30
Adenocarcinoma	25	31
Adenosquamous	1	1
Small cell	1	1
No biopsy	30	37
Size (mm)		
Median	24	
Range	3–52	
33rd Percentile	20	
66th Percentile	27	
T-Stage		
T1	61	75
T2	20	25
Location		
Central	21	26
Peripheral	60	74

Abbreviations: pts = patients; PS = performance status.

**Table 3:** Cox regression survival analysis for the impact of age, PS, TVI, histological status, and BED_α/β = 10_ to LPFS. (Chi-square = 20.31, *p* < 0.001).

	Univariate			Multivariate		
	*p*	HR	95% CI	*p*	HR	95% CI
Age	0.41	-	-	-	-	-
PS	0.014	0.28	0.07–0.49	0.003	0.34	0.16–0.37
TVI (cc)	0.217	-	-	-	-	-
Histological status	0.89	-	-	-	-	-
BED_α/β = 10_	0.033	1.061	1.009–1.089	-	-	-

Abbreviations: PS = performance status; TVI = irradiated tumor volume; BED = biologically effective dose; LPFS = local progression-free survival; HR = hazard ratio; CI = confidence interval.

**Table 4:** Regression analysis for the impact of age, PS, TVI, and BED_α/β = 3_ on the lung radiation-induced toxicity score. (F = 15.22, *p* < 0.001).

	Univariate			Multivariate		
	*p*	HR	95% CI	*p*	HR	95% CI
Age	0.44	-	-	-	-	-
PS	0.49	-	-	-	-	-
TVI (cc)	<0.001	4.02	3.61–4.83	<0.001	4.07	3.62–4.82
BED_α/β = 3_	<0.001	5.52	5.12–5.81	<0.001	5.62	5.11–5.83

Abbreviations: PS = performance status; TVI = irradiated tumor volume; BED = biologically effective dose; HR = hazard ratio; CI = confidence interval.

## Data Availability

Research data are stored in an institutional repository and will be shared upon reasonable request to the corresponding author.

## Supplementary Materials

The following supporting information can be downloaded at https://www.mdpi.com/article/10.3390/cancers16183227/s1: Table S1. Radiological Grading Scale of Radiation-Induced Lung Toxicity [23].

## References

[1] Siegel R.L., Giaquinto A.N., Jemal A. (2024). Cancer statistics, 2024. CA Cancer J. Clin..

[2] Mountzios G., Gkiozos I., Stratakos G., Pissakas G., Charpidou A., Toukfetzian L., Vamvakaris I., Syrigos K. (2021). Lung Cancer in Greece. J. Thorac. Oncol..

[3] Molina J.R., Yang P., Cassivi S.D., Schild S.E., Adjei A.A. (2008). Non-small cell lung cancer: Epidemiology, risk factors, treatment, and survivorship. Mayo Clin. Proc..

[4] Lagerwaard F.J., Verstegen N.E., Haasbeek C.J., Slotman B.J., Paul M.A., Smit E.F., Senan S. (2012). Outcomes of stereotactic ablative radiotherapy in patients with potentially operable stage I non-small cell lung cancer. Int. J. Radiat. Oncol. Biol. Phys..

[5] Chang J.Y., Senan S., Paul M.A., Mehran R.J., Louie A.V., Balter P., Groen H.J., McRae S.E., Widder J., Feng L. (2015). Stereotactic ablative radiotherapy versus lobectomy for operable stage I non-small-cell lung cancer: A pooled analysis of two randomised trials. Lancet Oncol..

[6] Verstegen N.E., Oosterhuis J.W., Palma D.A., Rodrigues G., Lagerwaard F.J., van der Elst A., Mollema R., van Tets W.F., Warner A., Joosten J.J. (2013). Stage I-II non-small-cell lung cancer treated using either stereotactic ablative radiotherapy (SABR) or lobectomy by video-assisted thoracoscopic surgery (VATS): Outcomes of a propensity score-matched analysis. Ann. Oncol..

[7] Bogart J.A., Hodgson L., Seagren S.L., Blackstock A.W., Wang X., Lenox R., Turrisi A.T., Reilly J., Gajra A., Vokes E.E. (2010). Phase I study of accelerated conformal radiotherapy for stage I non-small-cell lung cancer in patients with pulmonary dysfunction: CALGB 39904. J. Clin. Oncol..

[8] Cheung P., Faria S., Ahmed S., Chabot P., Greenland J., Kurien E., Mohamed I., Wright J.R., Hollenhorst H., de Metz C. (2014). Phase II study of accelerated hypofractionated three-dimensional conformal radiotherapy for stage T1-3 N0 M0 non-small cell lung cancer: NCIC CTG BR.25. J. Natl. Cancer Inst..

[9] Nyman J., Hallqvist A., Lund J.A., Brustugun O.T., Bergman B., Bergstrom P., Friesland S., Lewensohn R., Holmberg E., Lax I. (2016). SPACE—A randomized study of SBRT vs. conventional fractionated radiotherapy in medically inoperable stage I NSCLC. Radiother. Oncol..

[10] Ball D., Mai G.T., Vinod S., Babington S., Ruben J., Kron T., Chesson B., Herschtal A., Vanevski M., Rezo A. (2019). Stereotactic ablative radiotherapy versus standard radiotherapy in stage 1 non-small-cell lung cancer (TROG 09.02 CHISEL): A phase 3, open-label, randomised controlled trial. Lancet Oncol..

[11] Ding C., Chang C.H., Haslam J., Timmerman R., Solberg T. (2010). A dosimetric comparison of stereotactic body radiation therapy techniques for lung cancer: Robotic versus conventional linac-based systems. J. Appl. Clin. Med. Phys..

[12] Verma V., Simone C.B., Allen P.K., Gajjar S.R., Shah C., Zhen W., Harkenrider M.M., Hallemeier C.L., Jabbour S.K., Matthiesen C.L. (2017). Multi-Institutional Experience of Stereotactic Ablative Radiation Therapy for Stage I Small Cell Lung Cancer. Int. J. Radiat. Oncol. Biol. Phys..

[13] Shioyama Y., Onishi H., Takayama K., Matsuo Y., Takeda A., Yamashita H., Miyakawa A., Murakami N., Aoki M., Matsushita H. (2018). Clinical Outcomes of Stereotactic Body Radiotherapy for Patients With Stage I Small-Cell Lung Cancer: Analysis of a Subset of the Japanese Radiological Society Multi-Institutional SBRT Study Group Database. Technol. Cancer Res. Treat..

[14] Chang J.Y., Mehran R.J., Feng L., Verma V., Liao Z., Welsh J.W., Lin S.H., O’Reilly M.S., Jeter M.D., Balter P.A. (2021). Stereotactic ablative radiotherapy for operable stage I non-small-cell lung cancer (revised STARS): Long-term results of a single-arm, prospective trial with prespecified comparison to surgery. Lancet Oncol..

[15] Moghanaki D., Karas T., Timmerman R.D., Cameron R.B., Ritter T.A., Shi H., Leiner M.K., Feng H., Skinner V.L., Robin L. (2023). Protocol for the Veterans Affairs Cooperative Studies Program Study Number 2005: A Phase 3 Randomized Trial of Lung Cancer Surgery or Stereotactic Radiotherapy for Operable Early-Stage Non-Small Cell Lung Cancer. CHEST Pulm..

[16] Mehta N., King C.R., Agazaryan N., Steinberg M., Hua A., Lee P. (2012). Stereotactic body radiation therapy and 3-dimensional conformal radiotherapy for stage I non-small cell lung cancer: A pooled analysis of biological equivalent dose and local control. Pract. Radiat. Oncol..

[17] Song C.W., Kim M.S., Cho L.C., Dusenbery K., Sperduto P.W. (2014). Radiobiological basis of SBRT and SRS. Int. J. Clin. Oncol..

[18] Bentzen S.M., Skoczylas J.Z., Bernier J. (2000). Quantitative clinical radiobiology of early and late lung reactions. Int. J. Radiat. Biol..

[19] Klement R.J., Sonke J.J., Allgauer M., Andratschke N., Appold S., Belderbos J., Belka C., Dieckmann K., Eich H.T., Flentje M. (2020). Estimation of the alpha/beta ratio of non-small cell lung cancer treated with stereotactic body radiotherapy. Radiother. Oncol..

[20] Eisenhauer E.A., Therasse P., Bogaerts J., Schwartz L.H., Sargent D., Ford R., Dancey J., Arbuck S., Gwyther S., Mooney M. (2009). New response evaluation criteria in solid tumours: Revised RECIST guideline (version 1.1). Eur. J. Cancer.

[21] O J.H., Lodge M.A., Wahl R.L. (2016). Practical PERCIST: A Simplified Guide to PET Response Criteria in Solid Tumors 1.0. Radiology.

[22] Common Terminology Criteria for Adverse Events (CTCAE) Version 5.0, Cancer therapy Evaluation Program, Division of Cancer Treatment and Diagnosis, NIH/National Cancer Institute. https://ctep.cancer.gov/protocoldevelopment/electronic_applications/docs/CTCAE_v5_Quick_Reference_5x7.pdf.

[23] Kouloulias V., Zygogianni A., Efstathopoulos E., Victoria O., Christos A., Pantelis K., Koutoulidis V., Kouvaris J., Sandilos P., Varela M. (2013). Suggestion for a new grading scale for radiation induced pneumonitis based on radiological findings of computerized tomography: Correlation with clinical and radiotherapeutic parameters in lung cancer patients. Asian Pac. J. Cancer Prev..

[24] Johnson R.A., Wichern D.W. (2007). Applied Multivariate Statistical Analysis.

[25] Chen H., Laba J.M., Boldt R.G., Goodman C.D., Palma D.A., Senan S., Louie A.V. (2018). Stereotactic Ablative Radiation Therapy Versus Surgery in Early Lung Cancer: A Meta-analysis of Propensity Score Studies. Int. J. Radiat. Oncol. Biol. Phys..

[26] Vlaskou Badra E., Baumgartl M., Fabiano S., Jongen A., Guckenberger M. (2021). Stereotactic radiotherapy for early stage non-small cell lung cancer: Current standards and ongoing research. Transl. Lung Cancer Res..

[27] Khadige M., Salleron J., Marchesi V., Oldrini G., Peiffert D., Beckendorf V. (2018). Cyberknife((R)) stereotactic radiation therapy for stage I lung cancer and pulmonary metastases: Evaluation of local control at 24 months. J. Thorac. Dis..

[28] Kocak Uzel E., Bagci Kilic M., Morcali H., Uzel O. (2023). Stereotactic body radiation therapy for stage I medically operable non-small cell lung cancer. Sci. Rep..

[29] Stanic K., But-Hadzic J., Zagar J., Vrankar M. (2023). Local control and survival after stereotactic body radiation therapy of early-stage lung cancer patients in Slovenia. Radiol. Oncol..

[30] Sun B., Brooks E.D., Komaki R.U., Liao Z., Jeter M.D., McAleer M.F., Allen P.K., Balter P.A., Welsh J.D., O’Reilly M.S. (2017). 7-year follow-up after stereotactic ablative radiotherapy for patients with stage I non-small cell lung cancer: Results of a phase 2 clinical trial. Cancer.

[31] Gensheimer M.F., Gee H., Shirato H., Taguchi H., Snyder J.M., Chin A.L., Vitzthum L.K., Maxim P.G., Wakelee H.A., Neal J. (2023). Individualized Stereotactic Ablative Radiotherapy for Lung Tumors: The iSABR Phase 2 Nonrandomized Controlled Trial. JAMA Oncol..

[32] Kang J., Ning M.S., Feng H., Li H., Bahig H., Brooks E.D., Welsh J.W., Ye R., Miao H., Chang J.Y. (2020). Predicting 5-Year Progression and Survival Outcomes for Early Stage Non-small Cell Lung Cancer Treated with Stereotactic Ablative Radiation Therapy: Development and Validation of Robust Prognostic Nomograms. Int. J. Radiat. Oncol. Biol. Phys..

[33] Song C.W., Glatstein E., Marks L.B., Emami B., Grimm J., Sperduto P.W., Kim M.S., Hui S., Dusenbery K.E., Cho L.C. (2021). Biological Principles of Stereotactic Body Radiation Therapy (SBRT) and Stereotactic Radiation Surgery (SRS): Indirect Cell Death. Int. J. Radiat. Oncol. Biol. Phys..

[34] Cases C., Benegas M., Sanchez M., Vollmer I., Casas F., Goma C., Molla M. (2023). Biological equivalent dose is associated with radiological toxicity after lung stereotactic ablative radiation therapy. Radiother. Oncol..

[35] Borelli C., Vergara D., Simeone A., Pazienza L., Castorani G., Graziano P., Di Micco C., Quarato C.M.I., Sperandeo M. (2022). CT-Guided Transthoracic Biopsy of Pulmonary Lesions: Diagnostic versus Nondiagnostic Results. Diagnostics.

[36] Dautruche A., Filion E., Mathieu D., Bahig H., Roberge D., Lambert L., Vu T., Campeau M.P. (2020). To Biopsy or Not to Biopsy?: A Matched Cohort Analysis of Early-Stage Lung Cancer Treated with Stereotactic Radiation with or without Histologic Confirmation. Int. J. Radiat. Oncol. Biol. Phys..

[37] Chang J.Y., Lin S.H., Dong W., Liao Z., Gandhi S.J., Gay C.M., Zhang J., Chun S.G., Elamin Y.Y., Fossella F.V. (2023). Stereotactic ablative radiotherapy with or without immunotherapy for early-stage or isolated lung parenchymal recurrent node-negative non-small-cell lung cancer: An open-label, randomised, phase 2 trial. Lancet.

