# Supplementary material for: Robotic Stereotactic Ablative Radiotherapy for Patients with Early-Stage Lung Cancer: Results of an Interim Analysis
Source: Cancers (Basel). 2024 Sep 22;16(18):3227. doi: 10.3390/cancers16183227 (PMC11429671; doi:10.3390/cancers16183227)
Supplement: Supplementary file 1 [file cancers-16-03227-s001.zip › cancers-3201618-supplementary.pdf]

Table S1. Radiological Grading Scale of Radiation-Induced Lung Toxicity **[23]**.

Grade	CT Findings
0	No Findings.
1	Ground glass opacities without fuzziness of the subjacent pulmonary vessels.
2	The findings may vary from ground glass opacities, extending beyond the radiation field, to consolidations.
3	Clear focal consolidation ± elements of fibrosis.
4	Dense consolidation, cicatrisation atelectasis, aerobronchogram and bronchial extension (traction bronchiectasis), significant pulmonary volume loss, and pleural thickening.
